# The liver–gut–bone axis in hepatic osteodystrophy: mechanisms, clinical evidence and therapeutic perspectives

**DOI:** 10.3389/fcimb.2026.1935323

**Published:** 2026-10-12

**Authors:** Liangping Zhang, Ting Jiang, Rongqi Cao, Linqiu Han, Ligang Ni

**Affiliations:** 1 Department of Orthopaedics, Hangzhou Linping District Hospital of Integrated Traditional Chinese and Western Medicine, Hangzhou, Zhejiang, China; 2 Department of Orthopaedics, The Second People’s Hospital of Lishui, Lishui, Zhejiang, China; 3 Department of Orthopaedics, Sir Run Run Shaw Hospital, Zhejiang University School of Medicine, Hangzhou, Zhejiang, China

**Keywords:** bile acids, chronic liver disease, gut microbiota, hepatic osteodystrophy, liver-gut-bone axis, osteoporosis

## Abstract

Hepatic osteodystrophy (HOD) is an important but under-recognized complication of chronic liver disease (CLD), characterized by reduced bone mass, impaired bone microarchitecture and increased fragility fracture risk. Its skeletal phenotype and pathogenic mechanisms vary substantially according to CLD aetiology and disease stage. In metabolic dysfunction-associated steatotic liver disease (MASLD), skeletal risk is closely associated with metabolic dysfunction and hepatic fibrosis; alcohol-associated liver disease combines direct ethanol-related osteotoxicity with malnutrition and endocrine abnormalities; viral hepatitis-associated bone disease is influenced by persistent inflammation, disease stage and antiviral exposure; and cholestatic liver diseases are characterized by disrupted bile acid homeostasis, fat-soluble vitamin malabsorption and impaired bone formation. The liver–gut–bone axis provides an integrative framework linking these disease-specific features. Gut dysbiosis, intestinal barrier dysfunction, microbial translocation and altered metabolites, including short-chain fatty acids, bile acids and tryptophan-derived metabolites, may contribute to abnormal bone remodelling through inflammatory, metabolic and nutritional pathways. Cirrhosis further amplifies skeletal injury through sarcopenia, malnutrition and endocrine dysfunction. However, human evidence establishing a complete causal liver–gut–bone pathway remains limited. Current management should therefore focus on treatment of the underlying liver disease, fracture-risk assessment, nutritional and lifestyle interventions and evidence-based anti-osteoporotic therapy, while microbiota- and metabolite-targeted strategies remain investigational. Future prospective studies integrating microbiome and metabolomic profiling with bone turnover, microarchitecture and fracture outcomes are needed to clarify causal mechanisms and improve individualized management of CLD-associated HOD.

## 1 Introduction

CLD encompasses a heterogeneous group of disorders characterized by persistent hepatic injury that may lead to inflammation, fibrosis, cirrhosis and hepatic dysfunction. Major aetiologies of CLD include MASLD, ALD, chronic viral hepatitis, autoimmune liver diseases and cholestatic liver diseases, all of which may progress to cirrhosis (Rinella et al., 2023). CLD is a major contributor to global morbidity and mortality, while the rising prevalence of obesity, type 2 diabetes and metabolic syndrome has particularly increased the burden of MASLD (Gan et al., 2025). In patients with cirrhosis, contemporary management focuses on treating the underlying aetiology and preventing or managing complications; however, reversal of advanced fibrosis and prevention of decompensation remain major clinical challenges (Tapper and Parikh, 2023). CLD may be complicated by HOD, a spectrum of metabolic bone disorders that predominantly includes osteoporosis and, less commonly, osteomalacia. Osteoporosis is a systemic skeletal disorder characterized by reduced bone mass, deterioration of bone microarchitecture and increased susceptibility to fragility fractures (Gao et al., 2024). Established and proposed contributors to CLD-associated bone loss include malnutrition, vitamin D insufficiency, hypogonadism, chronic inflammation, physical inactivity and sarcopenia, with their relative contributions varying according to liver-disease aetiology and stage (Saeki and Tsubota, 2021; Pramanik et al., 2025). Therefore, elucidating the mechanisms underlying CLD-associated bone loss and identifying new therapeutic strategies remain urgent priorities in this field.

As the number of patients with CLD increases, HOD, a CLD-associated metabolic bone disorder, has attracted growing attention. Clinically, HOD is most commonly manifested as reduced bone mineral density and increased susceptibility to fragility fractures (Jadzic and Djonic, 2023). Malnutrition and nutritional deficiencies are recognized contributors to skeletal impairment in CLD. In particular, inadequate nutritional status and vitamin D deficiency may adversely affect bone health through impaired calcium homeostasis and reduced bone formation, while coexisting sarcopenia may further increase skeletal fragility (Jeong and Kim, 2019). Although endocrine dysfunction, nutritional deficiency and chronic inflammation are central to the pathogenesis of HOD, these mechanisms do not fully explain its complex pathological features, particularly its clinical heterogeneity, early abnormalities in bone remodelling and the incomplete parallelism between hepatic injury and skeletal damage (Yoon et al., 2021; Lu et al., 2022). These observations suggest that interactions among established liver–bone, gut–liver and gut–bone pathways may collectively contribute to HOD pathogenesis, although a continuous causal liver–gut–bone pathway has not yet been demonstrated in humans (Han et al., 2026). Increasing evidence indicates that gut microbial dysbiosis and altered microbial metabolites may represent a critical link between the progression of liver injury and impaired bone remodelling (Hsu and Schnabl, 2023; You et al., 2025; Zhang et al., 2025).

The intestinal microecosystem is one of the most complex microbial ecosystems in the human body, containing approximately 3.8 × 10¹³ bacteria and a collective gene repertoire that greatly expands host metabolic and immunological capacity (Human Microbiome Project, 2012; Sender et al., 2016). The gut microbiota (GM) and its metabolites not only influence hepatic lipid accumulation and inflammatory responses but may also contribute to disordered bone metabolism by regulating intestinal barrier integrity and systemic chronic inflammation (Tilg et al., 2021; You et al., 2025). In this Review, we use the liver–gut–bone axis as an integrative framework to examine how gut microbial dysbiosis, hepatic metabolic and inflammatory abnormalities and disturbed bone remodelling may interact in CLD. This review provides an integrated overview of alterations in gut microbial composition and microbiota-derived metabolites in osteoporosis associated with CLD. It further discusses the possible molecular pathways involved in the liver–gut–bone axis and summarizes potential therapeutic approaches, thereby offering theoretical support for the clinical prevention and management of this condition.

For this narrative review, we conducted a structured literature search of PubMed, Web of Science and Embase for English-language studies published from database inception to August 2026. Search terms included combinations of “chronic liver disease”, “cirrhosis”, “MASLD”, “alcohol-associated liver disease”, “cholestatic liver disease”, “osteoporosis”, “bone mineral density”, “fracture”, “gut microbiota”, “intestinal barrier”, “bile acids”, “short-chain fatty acids” and “faecal microbiota transplantation”. Priority was given to systematic reviews, meta-analyses, clinical cohort studies and mechanistic studies directly relevant to CLD-associated skeletal disease. Experimental studies from non-CLD models were included when they provided mechanistic context not otherwise available. The search was intended to identify representative clinical and mechanistic evidence rather than to constitute a formal systematic review or quantitative evidence synthesis.

## 2 Clinical evidence for hepatic osteodystrophy across CLD subtypes

### 2.1 Definition and skeletal phenotypes

HOD is an umbrella term encompassing metabolic bone disorders associated with CLD, and is primarily characterized by osteopenia, osteoporosis and deterioration of bone microarchitecture; osteomalacia may also occur in some patients with cholestatic liver disease. Dual-energy X-ray absorptiometry (DXA) remains the standard method for assessing bone mineral density (BMD), although the results should be interpreted in the context of age, sex and the overall clinical setting. World Health Organization T-score thresholds are primarily applicable to postmenopausal women and men aged 50 years or older, whereas assessment in premenopausal women, men younger than 50 years and children should incorporate Z-scores, a history of fragility fractures and secondary risk factors. The Fracture Risk Assessment Tool (FRAX) can assist fracture-risk estimation in selected adults; however, because it was not specifically developed for patients with CLD, its estimates should be interpreted alongside liver disease aetiology and stage, sarcopenia and the risk of falls (Morin et al., 2023; Slart et al., 2025).

HOD is common among patients with CLD, and both its prevalence and severity generally increase with progression of liver disease. Low bone mass and osteoporosis are common among patients with advanced chronic liver disease, particularly those undergoing evaluation for liver transplantation (Thomson et al., 2024; Dove et al., 2026). A similarly high prevalence of low bone mass and osteoporosis has been observed in individuals with steatotic liver disease accompanied by metabolic dysfunction (Zhang et al., 2023). Although estimates vary across geographical regions, ethnic groups and study populations, greater hepatic fibrosis burden has been associated with impaired bone health, and higher Fibrosis-4 Index (FIB-4) values have been linked to an increased risk of osteoporosis in selected populations (Yu et al., 2022). With progression to cirrhosis, malnutrition, sarcopenia, hypogonadism, abnormalities in vitamin D metabolism and other systemic complications may further contribute to skeletal fragility (Guanabens and Pares, 2018).

Skeletal phenotypes differ substantially among CLD subtypes, not only in the extent of BMD reduction but also in patterns of microarchitectural deterioration and fracture. Cholestatic liver diseases, particularly primary biliary cholangitis (PBC), are frequently accompanied by marked bone loss, with reported osteoporosis prevalence ranging from 20% to 56%; skeletal sites rich in trabecular bone, such as the vertebrae, appear to be particularly vulnerable (Chen et al., 2023). By contrast, bone loss is generally less pronounced in patients with compensated chronic viral hepatitis, although the risk of low BMD increases substantially with greater viral activity, progression of hepatic fibrosis or transition to cirrhosis (Bering et al., 2018; Zhou et al., 2026). ALD can affect both trabecular and cortical bone, and experimental studies indicate that chronic alcohol exposure can impair trabecular microarchitecture and cortical bone quality while suppressing osteogenic activity (Liu et al., 2022; Shin et al., 2024). Thus, skeletal involvement across CLD subtypes cannot be adequately characterized as a uniform low-BMD phenotype but should instead be interpreted in relation to both disease aetiology and stage.

In addition to loss of bone mass, abnormalities in bone quality and bone turnover are important components of HOD. Patients with cirrhosis can exhibit deterioration in trabecular microarchitecture and proximal hip geometry, while vertebral fractures may occur despite BMD alone incompletely capturing skeletal fragility (Ogiso et al., 2022; Thakur et al., 2022). Among individuals with obesity and steatotic liver disease, deterioration in trabecular bone score (TBS) has also been associated with the severity of hepatic fibrosis (Barchetta et al., 2023). Bone turnover in HOD is strongly dependent on both aetiology and disease stage. Suppressed bone formation is among the more consistent features, whereas increased bone resorption may coexist in subsets of patients with cirrhosis or cholestatic liver disease. Bone marrow mesenchymal stem cells from patients with cirrhosis show impaired osteogenic capacity and reduced expression of osteogenesis-related markers, including RUNX2 and COL1A1, whereas bone resorption markers such as CTX and TRAcP5b may be elevated in some patients (Lu et al., 2022). In addition, impaired hepatic function can alter the metabolism and clearance of circulating markers such as procollagen type I N-terminal propeptide (P1NP) and sclerostin, while vitamin D deficiency may further complicate interpretation of bone turnover indices (Jorgensen et al., 2022). Clinical evaluation of HOD should therefore extend beyond BMD to incorporate disease aetiology and severity, bone microarchitecture, fracture history and bone turnover status, thereby providing a more robust basis for risk stratification and mechanistic analyses across CLD subtypes.

### 2.2 MASLD: skeletal risk

Clinical associations between MASLD and abnormalities in bone metabolism have been reported, although both the direction and magnitude of these associations vary across populations and are influenced by age, sex, menopausal status, obesity, type 2 diabetes, ethnicity and the severity of hepatic fibrosis. Current evidence more consistently suggests that advanced fibrosis is a stronger indicator of skeletal risk than steatosis alone. Among patients with type 2 diabetes and MASLD, a greater fibrosis burden has been associated with lower BMD and higher risks of osteopenia and osteoporosis (Pan et al., 2024). Longitudinal studies have similarly shown that higher FIB-4 scores in men with NAFLD or MASLD are associated with a more rapid decline in total hip BMD during follow-up, while hepatic fibrosis in individuals with obesity and NAFLD has been linked to impaired bone mineralization and deterioration of bone microarchitecture (Yoon et al., 2021; Barchetta et al., 2023). Overall, the association between MASLD and skeletal impairment remains heterogeneous across populations.

The heterogeneity of MASLD-associated bone disease is further shaped by metabolic comorbidities such as obesity, insulin resistance and type 2 diabetes, each of which can independently alter bone turnover and fracture risk (Chondrogianni et al., 2024). Insulin resistance, chronic low-grade inflammation, adipokine dysregulation and alterations in the gut microbiota and microbial metabolites may all contribute to skeletal injury, although direct causal evidence in humans remains limited for many of these mechanistic pathways (Tilg et al., 2021; Hofbauer et al., 2022; Chen et al., 2026). In particular, impaired intestinal barrier integrity and translocation of microbial products, together with alterations in microbiota-derived metabolites such as short-chain fatty acids and bile acids, provide biologically plausible pathways linking MASLD to dysregulated bone remodelling (Hsu and Schnabl, 2023; Herrmann et al., 2024). MASLD-associated HOD should therefore be viewed as a complex phenotype jointly determined by the severity of liver disease and the host metabolic milieu, and its clinical assessment should place particular emphasis on fibrosis stage, metabolic comorbidities and established fracture risk factors.

### 2.3 ALD: bone loss and fractures

ALD is an important cause of HOD, in which skeletal injury is driven by a combination of direct alcohol-related osteotoxicity, nutritional and endocrine disturbances, and progression of liver disease. Ethanol and its metabolite acetaldehyde can suppress osteoblast proliferation and differentiation while promoting osteoclast-associated activity, thereby shifting bone remodelling towards net bone loss (Chen et al., 2011; Hoshi et al., 2012). At the same time, patients with ALD frequently exhibit protein–energy malnutrition, vitamin D deficiency, hypogonadism and sarcopenia, all of which further increase skeletal fragility (Seitz et al., 2018). Alcohol-induced gut microbial dysbiosis and impaired intestinal barrier function may also facilitate the translocation of microbial products such as lipopolysaccharide, resulting in Kupffer-cell activation and systemic inflammatory responses and thereby providing a potential mechanistic link between hepatic injury and abnormal bone metabolism (Szabo, 2015). Nevertheless, evidence linking these mechanisms directly to skeletal outcomes in humans remains limited. ALD-associated HOD should therefore be regarded as a clinical phenotype arising from the combined effects of direct alcohol toxicity and secondary consequences of liver disease.

Clinical studies indicate that skeletal risk in ALD extends beyond reductions in BMD to include deterioration of bone microarchitecture and an increased fracture burden. In a nationwide cohort of 25, 090 patients with alcohol-associated cirrhosis, fracture incidence was substantially higher than that in the general population, and fractures were associated with further increases in 30-day and 1-year mortality (Wester et al., 2023). In patients with cirrhosis, reduced lumbar trabecular bone score and deterioration of peripheral trabecular and cortical microarchitecture have been documented, indicating alterations in bone quality in addition to reductions in areal BMD (Wakolbinger et al., 2019). Animal studies further demonstrate that chronic ethanol consumption induces osteopenia through activation of osteoblast necroptosis, leading to reduced bone mass and deterioration of trabecular architecture (Luo et al., 2017; Guo et al., 2021). Collectively, human microarchitectural studies support the concept that skeletal risk in ALD reflects both direct alcohol-related osteotoxicity and the severity of liver disease, with substantial vertebral trabecular deterioration indicating a bone-quality defect beyond BMD alone (Jadzic et al., 2020). Accordingly, assessment of bone health in ALD should integrate BMD, previous fragility fractures, bone quality, fall risk, nutritional status and liver disease severity rather than relying on BMD as a single metric.

### 2.4 Viral hepatitis: skeletal outcomes

The clinical associations of chronic hepatitis B (CHB) and chronic hepatitis C (CHC) with bone loss are heterogeneous, and studies have yielded inconsistent findings regarding both BMD and fracture risk. Some studies have reported only modest differences in BMD between patients with CHB or CHC and healthy controls, whereas substantially higher prevalences of osteopenia and osteoporosis have been observed in populations with coinfection, prolonged antiviral exposure or other high-risk features. For example, among individuals with HIV/HCV coinfection, the baseline prevalence of osteoporosis at the lumbar spine and femoral neck was 17.6% and 7.2%, respectively, yet BMD did not improve significantly after achievement of a sustained virological response (SVR) (Carrero et al., 2021). In another study including individuals with HIV, HBV or HCV infection, the prevalence of osteoporosis and osteopenia was 14% and 48%, respectively, and treatment regimens containing tenofovir or protease inhibitors were associated with vertebral fracture risk (Dalla Grana et al., 2019). Beyond bone mass, HCV infection may also affect bone quality: studies using TBS have identified an independent association between HCV infection and deterioration of lumbar trabecular microarchitecture, and volumetric BMD assessed by quantitative computed tomography (CT) was lower in postmenopausal women with HIV/HCV coinfection than in those with HIV monoinfection (Bedimo et al., 2018; T Yin et al., 2018). Thus, viral hepatitis-associated bone disease is not determined by viral replication alone but is also shaped by age, sex, immune status, coinfection and treatment exposure. In chronic HBV infection, a higher viral burden has been associated with lower BMD in antiviral-naïve patients, whereas skeletal responses following HCV cure remain variable across cohorts. These observations underscore the importance of explicitly reporting virological status, antiviral exposure and fibrosis stage when interpreting skeletal outcomes (Lampertico et al., 2020; Li et al., 2024; Lo Re et al., 2025).

Antiviral therapy adds a further layer of complexity to studies of bone metabolism in viral hepatitis. Compared with the interferon–ribavirin era, direct-acting antivirals (DAAs) substantially reduce the inflammatory burden associated with HCV infection; however, current evidence does not indicate a parallel short-term recovery of BMD after viral clearance. In one prospective study of individuals with HIV/HCV coinfection, HCV cure was accompanied by marked reductions in inflammatory markers, including soluble TNF receptor 1 (sTNFR-1), sTNFR-2 and sCD163, whereas BMD at the lumbar spine, femoral neck and total hip, as well as TBS, remained unchanged. By contrast, the bone formation marker P1NP increased, suggesting that improvement in bone remodelling may precede detectable changes in bone mass (Psomas et al., 2025). Data from the Swiss HIV Cohort Study likewise showed that HCV seropositivity was associated with increased risks of osteoporosis and fracture, but that these risks were not entirely dependent on persistent HCV RNA positivity (Kovari et al., 2017). Collectively, these findings suggest that viral eradication and attenuation of inflammation do not necessarily translate into immediate gains in BMD; skeletal recovery may be delayed and may remain influenced by pre-existing liver injury, treatment exposure and the host metabolic milieu.

At more advanced stages of disease, skeletal injury associated with viral hepatitis is more likely to reflect the combined effects of hepatic dysfunction, nutritional and endocrine abnormalities, and chronic inflammation than a direct effect of the virus alone. Cirrhosis can be accompanied by reduced synthesis of vitamin D-binding protein and insulin-like growth factor 1 (IGF-1), impaired absorption of fat-soluble vitamins and minerals, and hypogonadism, all of which can suppress bone formation and increase skeletal fragility. However, human studies have shown inconsistent associations between the severity of hepatic fibrosis and either BMD or TBS, indicating that skeletal risk cannot be explained by fibrosis burden alone. Experimental studies have proposed direct effects of HCV-related proteins on osteoclastogenic signalling, but clinical evidence supporting such a pathway remains limited. In humans, improvement in systemic inflammation after HCV cure does not necessarily translate into short-term improvement in BMD or TBS (Psomas et al., 2025). The clinical relevance of this pathway therefore remains uncertain. HCV-associated chronic inflammation and alterations in sex-hormone regulation may also contribute to abnormal bone metabolism; for example, higher sex hormone-binding globulin concentrations have been associated with lower lumbar-spine BMD and poorer physical function in women (Dias et al., 2022). Overall, HOD associated with viral hepatitis is strongly dependent on host characteristics, disease stage and treatment exposure. Clinical studies and risk assessments should therefore report virological status, fibrosis severity, coinfection and antiviral treatment exposure to avoid attributing complex secondary skeletal injury solely to the viral infection itself.

### 2.5 Cholestatic liver disease: bone loss

PBC and primary sclerosing cholangitis (PSC) are representative cholestatic liver diseases associated with a high risk of bone loss, with the clinical evidence being more extensive for PBC. Low BMD is common in PBC, with prevalence estimates of 35–50% in some studies, and bone loss can occur relatively early in the disease course, suggesting that cholestasis-related factors may contribute to skeletal injury independently of advanced liver dysfunction (Pugliese et al., 2022). Patients with PSC are likewise at increased risk of osteoporosis, with skeletal risk further influenced by coexisting inflammatory bowel disease, low body weight and nutritional or fat-soluble vitamin deficiencies (Zakharia et al., 2018; Bowlus et al., 2023). In addition, studies in PSC have shown that increased frequencies of T helper 17 (Th17) cells are associated with higher concentrations of the bone resorption marker deoxypyridinoline and lower BMD, suggesting a potential contribution of immune-mediated bone resorption to skeletal loss (Schmidt et al., 2019). Thus, HOD in cholestatic liver disease does not represent a single uniform phenotype but rather reflects the combined effects of the severity of cholestasis, immune status and associated comorbidities.

The effects of cholestasis on bone metabolism predominantly involve impaired absorption of fat-soluble vitamins, dysregulated bile acid signalling and suppression of bone formation. Reduced delivery of bile acids to the intestinal lumen can compromise the absorption of dietary fat and fat-soluble vitamins, including vitamins D and K. Vitamin D deficiency can impair intestinal calcium absorption and bone mineralisation, whereas vitamin K deficiency may interfere with osteocalcin γ-carboxylation and compromise bone matrix quality; however, the relative contribution of these abnormalities to osteoporosis in cholestatic liver disease varies according to disease stage and nutritional status (Guanabens and Pares, 2018; Pugliese et al., 2022). Beyond their role in enterohepatic circulation, bile acids can modulate osteoblast and osteoclast function through signalling pathways involving the farnesoid X receptor (FXR) and Takeda G protein-coupled receptor 5 (TGR5). Disturbances in bile acid metabolism may also be accompanied by changes in gut microbial composition, potentially exerting additional effects on bone remodelling (Li et al., 2017; Zeng et al., 2023). Cholestatic liver disease therefore represents a particularly high-risk setting in which impaired bile flow can affect both bone formation and mineralization. In a rat model of bile duct ligation, cholestasis induced skeletal fragility and impaired osteoblastogenesis, providing disease-specific experimental support for suppressed bone formation (Weinreb et al., 2004). Although these findings strengthen the biological plausibility of impaired osteogenesis in cholestasis, this preclinical evidence should be distinguished from direct clinical evidence in humans.

PSC-associated bone disease is particularly dependent on the broader clinical context. In patients with concomitant inflammatory bowel disease, chronic inflammation, nutritional and vitamin D deficiencies, low body weight and exposure to glucocorticoids can further increase the risk of bone loss (European Association for the Study of the, 2022). Importantly, glucocorticoids are not routinely indicated for isolated PSC; their skeletal effects are therefore most relevant in patients with coexisting inflammatory bowel disease, autoimmune hepatitis overlap syndromes or other established indications for glucocorticoid therapy. Clinical observations implicating Th17 cells in PSC, together with experimental studies of IL-17 signalling in Abcb4-deficient mice, provide disease-specific support for a contribution of immune-mediated bone resorption (Schmidt et al., 2019). Overall, skeletal risk in cholestatic liver disease is strongly aetiology-dependent, and clinical assessment should integrate the severity of cholestasis, BMD and fracture risk, nutritional status, inflammatory bowel disease activity and medication exposure rather than relying on a single marker of hepatic function.

### 2.6 Cirrhosis: skeletal deterioration

Cirrhosis represents the common advanced stage of multiple forms of CLD and is closely associated with osteoporosis, deterioration of bone microarchitecture and an increased risk of fragility fractures. Studies using high-resolution peripheral quantitative computed tomography (HR-pQCT) have demonstrated impairment of both trabecular and cortical microarchitecture at the radius and tibia in patients with cirrhosis, with some abnormalities occurring independently of the severity of liver disease (Wakolbinger et al., 2019). Among patients undergoing evaluation for liver transplantation, CT imaging can additionally identify clinically unrecognized vertebral fractures, and low CT attenuation at the L1 vertebral body is strongly associated with prevalent vertebral fracture (Nachef et al., 2024). As CLD progresses to cirrhosis, the evidence for osteoporosis, microarchitectural deterioration and fragility fractures becomes more compelling, with human studies documenting abnormalities in both trabecular and cortical compartments together with clinically relevant vertebral fracture risk (Wakolbinger et al., 2019). Cirrhosis-associated bone loss is typically driven by multiple interacting factors, including malnutrition, sarcopenia, hypogonadism, abnormalities in vitamin D and calcium metabolism, reduced IGF-1 concentrations and chronic inflammation (Jeong and Kim, 2019; Mendez-Guerrero et al., 2024). Cirrhosis is therefore best regarded as a stage-dependent modifier that amplifies skeletal injury arising from different CLD aetiologies rather than as a single aetiology-independent mechanism of bone disease. In liver transplant candidates and recipients, the additional effects of disease severity, reduced physical activity, nutritional status and immunosuppressive therapy on changes in bone mass should also be considered.

### 2.7 Limitations of the clinical evidence

The current clinical evidence for HOD is characterized by substantial heterogeneity, which limits direct comparisons and quantitative synthesis across CLD subtypes. Evidence is particularly sparse for less common CLD subtypes such as hereditary haemochromatosis and Wilson disease, for which available clinical and mechanistic studies remain comparatively limited (Chenbhanich et al., 2018; Baschant et al., 2022; Zhu et al., 2025). In addition, studies differ considerably in patient selection, liver disease staging, methods used to assess BMD and definitions of skeletal outcomes. Because steatotic liver disease encompasses a spectrum from isolated steatosis to steatohepatitis and progressive fibrosis, careful stratification by disease stage is important when interpreting skeletal associations, which remain substantially heterogeneous across studies (Su et al., 2023). Most available studies are also cross-sectional, precluding robust assessment of the temporal sequence and causal relationship between liver disease progression and bone loss. Consequently, some of the observed associations may reflect shared risk factors, including metabolic dysfunction and chronic inflammation, rather than an independent effect of CLD itself.

Assessment of skeletal outcomes in the existing literature also has important limitations. Although BMD is the most widely used measure in studies of HOD, it does not fully capture bone microarchitecture, bone quality or mechanical strength. Clinically relevant vertebral fractures may occur in patients with cirrhosis even when DXA-based assessment does not fully indicate severe skeletal impairment, suggesting that reliance on BMD alone may underestimate fracture risk (Wakolbinger et al., 2019; Nachef et al., 2024). Nevertheless, data on fracture outcomes, bone-turnover phenotypes and high-resolution measures of bone microarchitecture remain comparatively limited across several forms of chronic liver disease, particularly outside cirrhosis and cholestatic liver disease (Jeong and Kim, 2019). Moreover, bone metabolism is strongly influenced by age, sex, menopausal status, body mass index, diabetes, nutritional status and medication exposure. Type 2 diabetes can increase skeletal fragility despite normal or increased BMD (Hofbauer et al., 2022), whereas alcohol-associated liver disease is additionally influenced by malnutrition, hypogonadism and direct alcohol-related osteotoxicity (Guanabens and Pares, 2018; Denys et al., 2022), making it difficult to isolate liver disease-specific effects from these overlapping determinants. Future studies will therefore require more rigorous patient stratification and multivariable adjustment to minimize confounding in comparisons across CLD subtypes.

More importantly, human clinical evidence supporting a complete liver–gut–bone axis remains limited. The available evidence consequently supports associations among CLD, gut microbial dysbiosis and skeletal injury more strongly than it supports a direct causal chain linking all three components. Future studies should prioritize prospective longitudinal designs stratified by CLD aetiology and disease stage, while simultaneously integrating microbiome and metabolomic profiling with measures of bone turnover, BMD, bone microarchitecture and fracture outcomes. Such integrated analyses will be essential for distinguishing causal and disease-specific components of the proposed liver–gut–bone axis from merely associative changes, establishing a more robust hierarchy of evidence and identifying mechanisms with genuine potential for clinical translation (**Table 1**).

**Table 1:** Representative human clinical evidence for skeletal abnormalities across major CLD subtypes and relevance to the liver–gut–bone axis.

CLD subtype	Study design	Population	Key assessments	Main findings	Axis relevance	Ref
MASLD	Retrospective longitudinal	2, 623 adults; 888 with NAFLD; 7.1 years	Serial BMD; FIB-4	Higher FIB-4 linked to faster hip BMD loss in men	Liver–bone; gut not assessed	(Yoon et al., 2021)
MASLD	Cross-sectional	1, 872 adults with obesity	DXA, TBS; FIB-4, osteocalcin, IGF-1	Higher FIB-4 linked to lower BMD and TBS	Liver fibrosis–bone; gut not assessed	(Barchetta et al., 2023)
ALD (cirrhosis)	Nationwide cohort	25, 090 cases; 239, 458 controls	Fractures; mortality	Higher fracture and post-fracture mortality risks	Cirrhosis–bone; gut not assessed	(Wester et al., 2023)
Viral hepatitis (CHB)	Cross-sectional	362 antiviral-naïve patients	DXA; β-CTX, CRP, HBV DNA	Higher HBV DNA linked to lower BMD	HBV–bone; gut not assessed	(Zhou et al., 2026)
Viral hepatitis (HCV)	Prospective cohort	59 achieving SVR; 53 controls	DXA, TBS, P1NP, CTX; inflammation	Inflammation decreased; BMD/TBS unchanged	Treatment/inflammation–bone; gut not assessed	(Psomas et al., 2025)
Cholestatic disease (PSC)	Translational study	PSC clinical cohort	DXA, HR-pQCT; Th17, resorption markers	Higher Th17 linked to greater resorption and lower bone mass	Immune–bone; gut not assessed	(Schmidt et al., 2019)
Cholestatic disease (PBC)	Population-based cohort	3, 980 cases; 37, 196 controls	Fractures; mortality	Higher osteoporotic fracture and post-fracture mortality risks	Cholestasis–bone; gut not assessed	(Schonau et al., 2023)
Cirrhosis	Retrospective study	376 LT candidates	L1 CT attenuation; vertebral fractures	Vertebral fractures associated with lower L1 attenuation	Advanced liver disease–bone	(Nachef et al., 2024)
Cirrhosis	Case-control	59 cases; 20 controls	P1NP, CTX, TRAcP5b, OPG, sclerostin	Bone resorption increased	Cirrhosis–bone turnover	(Jorgensen et al., 2022)

## 3 Mechanisms of the liver–gut–bone axis across CLD subtypes

### 3.1 MASLD: metabolic dysfunction

MASLD and its progressive form, metabolic dysfunction-associated steatohepatitis (MASH), are closely associated with the development of HOD, with upstream mechanisms involving gut microbial dysbiosis, impaired intestinal barrier integrity and metabolic inflammation. Patients with MASLD may exhibit depletion of short-chain fatty acid (SCFA)-producing taxa, including Roseburia and Faecalibacterium, together with enrichment of some lipopolysaccharide (LPS)-producing Gram-negative bacteria (Gupta et al., 2022). These inflammatory signals may subsequently reach the bone microenvironment and enhance RANKL–RANK signalling in osteoclast precursors, promoting osteoclast differentiation and resorptive activity, while TNF-α/IL-6-associated inflammatory signalling may impair osteoblast differentiation, matrix synthesis and mineralisation (Lyu et al., 2023). The resulting uncoupling of bone resorption from bone formation shifts bone remodelling towards net skeletal loss. Consistent with this model, high-fat feeding in experimental systems relevant to MASLD disrupts intestinal epithelial architecture, weakens tight-junction integrity and increases intestinal permeability; however, human evidence directly linking these barrier abnormalities to skeletal outcomes remains limited (Gupta et al., 2022). Thus, the sequence of dysbiosis, barrier dysfunction and inflammatory activation in MASLD is biologically plausible, but a complete causal liver–gut–bone pathway has yet to be demonstrated directly in humans.

Beyond inflammatory pathways, insulin resistance, adipokine dysregulation and alterations in microbial metabolites may jointly contribute to MASLD-associated skeletal injury. Insulin resistance and metabolic dysfunction may contribute to altered bone remodelling in MASLD, although the pathways involved are multifactorial. Clinically, greater hepatic fibrosis in individuals with obesity and NAFLD has been associated with impaired bone mineralization and bone microarchitecture (Barchetta et al., 2023). At the same time, depletion of butyrate-producing bacteria may attenuate the anti-inflammatory and bone-protective effects of SCFAs mediated through G-protein-coupled receptors such as GPR41 and GPR43 (Lyu et al., 2023; Wang et al., 2026). In clinical settings, skeletal risk in MASLD is additionally modified by obesity, diabetes and medication exposure; for example, thiazolidinediones may further increase the risk of bone loss (Zhang et al., 2021). Importantly, however, metabolic abnormalities in MASLD cannot be attributed exclusively to the gut microbiota. Recent experimental evidence indicates that bile acid abnormalities in MASH can also arise through host-driven, microbiota-independent mechanisms. In high-fat-fed *foz/foz* mice and an independent Western/high-fructose diet model, hepatic 7α-rehydroxylation contributed to reductions in secondary bile acids, cautioning against attributing all metabolite alterations to dysbiosis (Gillard et al., 2024). MASLD-associated HOD should therefore be understood as the product of interactions among the gut microbial ecosystem, host metabolism, insulin resistance and systemic inflammation rather than as a consequence of dysbiosis alone (Barchetta et al., 2023; Chondrogianni et al., 2024; Zhang et al., 2025).

### 3.2 ALD: dysbiosis and osteotoxicity

Skeletal injury in ALD displays relatively distinct disease-specific features, with upstream mechanisms centred on gut microbial dysbiosis, disruption of the intestinal barrier and amplification of gut-derived inflammation. Chronic alcohol consumption can alter gut microbial composition, characterized by increased relative abundance of Proteobacteria, depletion of selected commensal taxa, expansion of Gram-negative bacteria and an increased LPS burden (Jew and Hsu, 2023). In parallel, ethanol and its metabolite acetaldehyde can disrupt epithelial tight junctions and increase intestinal permeability, thereby facilitating the portal translocation of microbial products such as LPS (Cheng et al., 2025). These products can subsequently activate hepatic innate immune and inflammatory pathways, promoting the release of inflammatory mediators including TNF-α and IL-6 and exacerbating hepatic inflammation and fibrosis (Magdaleno et al., 2017). Chronic alcohol exposure also affects the intestinal mycobiome, promoting fungal overgrowth; fungal-derived β-glucans entering the circulation may further amplify liver injury through CLEC7A-dependent immune signalling (Yang et al., 2017). Human and translational evidence has additionally identified cytolysin-positive Enterococcus faecalis as a disease-relevant microbial feature of alcohol-associated hepatitis, and bacteriophage-mediated targeting of cytolytic E. faecalis attenuated ethanol-induced liver injury in humanized mouse models (Duan et al., 2019). Collectively, these findings provide relatively strong support for a disease-specific link among microbial dysbiosis, barrier dysfunction and hepatic inflammation in ALD, although direct human evidence extending this pathway to skeletal outcomes remains limited.

Against this background, the direct skeletal toxicity of ethanol and acetaldehyde, together with impaired nutrient and mineral absorption, may translate liver–gut disturbances into dysregulated bone remodelling. Acetaldehyde can suppress osteoblast proliferation and differentiation, whereas chronic ethanol exposure may enhance osteoclastogenic activity, producing an imbalance between bone formation and resorption that favours net bone loss (Chen et al., 2011; Hoshi et al., 2012). Patients with ALD frequently exhibit gut-derived endotoxaemia and heightened systemic inflammation, and LPS together with its downstream inflammatory mediators may promote osteoclastogenesis through Toll-like receptor 4 (TLR4) while suppressing osteoblast function, thereby establishing a plausible pathological continuum from intestinal barrier injury to disordered bone metabolism. Ethanol and acetaldehyde may themselves weaken epithelial tight junctions, increase gut permeability and facilitate LPS entry into the portal circulation; the resulting hepatic and systemic inflammatory signals can inhibit osteoblast activity and promote osteoclast differentiation, extending the consequences of barrier dysfunction beyond endotoxaemia to bone remodelling (Lyu et al., 2023; Raya Tonetti et al., 2024). In addition, alcohol-associated malnutrition and inadequate calcium and vitamin D intake can further compromise bone mineralization, while alcohol may reduce intestinal Calbindin-D9k expression and impair calcium absorption (Butts et al., 2023). ALD-associated HOD is therefore best viewed as the combined consequence of intestinal barrier dysfunction and systemic inflammation, direct alcohol-related osteotoxicity and impaired nutritional absorption rather than as the result of endotoxaemia or nutritional deficiency in isolation.

### 3.3 Viral hepatitis: immune dysregulation

Chronic HBV and HCV infections can be accompanied by bone loss, but evidence for a liver–gut–bone axis in viral hepatitis remains predominantly indirect. Studies combining 16S rRNA sequencing and metabolomic profiling in patients with CHB have shown that gut microbial composition and metabolite profiles vary with disease progression and antiviral treatment and are accompanied by changes in peripheral immune status (Shen et al., 2023). A combined human and experimental study identified microbial alterations associated with histological liver injury in CHB and further showed that faecal microbiota from HBV-related cirrhosis aggravated CCl4-induced hepatic fibrosis in recipient mice, providing disease-context-specific but indirect support for microbiome-mediated causality (Wang et al., 2025). These findings support a relationship between gut microbial abnormalities and hepatic injury in viral hepatitis; however, studies that simultaneously assess microbial alterations, liver disease progression and skeletal outcomes within the same human cohort are lacking. A complete causal liver–gut–bone pathway therefore cannot yet be inferred.

Persistent systemic inflammation may represent one mechanism linking chronic viral hepatitis to altered bone remodelling. In this setting, a sustained pro-inflammatory milieu may influence skeletal homeostasis by activating inflammatory pathways involving TLRs and NF-κB and by perturbing the RANKL–RANK–osteoprotegerin system, thereby favouring osteoclast differentiation and bone resorption while impairing bone formation (Tsukasaki and Takayanagi, 2019; Zhu et al., 2024). Clinical observations after HCV eradication are consistent with a contributory, but not exclusive, role of inflammation: systemic inflammatory markers decrease substantially after viral cure, whereas BMD and TBS do not necessarily improve over the same period (Psomas et al., 2025). This dissociation suggests that attenuation of inflammation alone may be insufficient to reverse established skeletal abnormalities. Importantly, this inflammation-mediated model should be distinguished from a direct effect of viral proteins on bone cells. Robust human evidence demonstrating that HBV- or HCV-derived viral proteins directly drive osteoclastogenesis in CLD is lacking. Proposed direct skeletal effects, such as induction of RANKL expression by the HCV core protein, are supported primarily by *in vitro* and experimental studies and should therefore be regarded as exploratory mechanisms rather than established clinical causal pathways. Overall, skeletal injury in viral hepatitis is more likely to reflect the combined influence of persistent inflammation, hepatic dysfunction, nutritional status, coinfection and treatment exposure.

Antiviral therapy is itself an important modifier that should be considered separately when evaluating skeletal health in viral hepatitis. In CHB, long-term treatment with tenofovir disoproxil fumarate (TDF) can be associated with renal tubular dysfunction, hypophosphataemia and adverse skeletal effects, warranting monitoring in the context of baseline renal function and fracture risk (Yip et al., 2024). Bone and renal safety should therefore be incorporated into the selection of long-term nucleos(t)ide analogue therapy. In a randomized phase III trial, tenofovir alafenamide maintained antiviral efficacy while producing smaller reductions in hip and spine BMD than TDF, supporting preferential consideration of agents with more favourable bone and renal safety profiles in patients at elevated skeletal or renal risk (Chan et al., 2016). In HCV infection, achievement of an SVR with direct-acting antiviral therapy substantially reduces systemic inflammation, but improvement in BMD over the short to medium term remains inconsistent (Psomas et al., 2025). Evaluation of viral hepatitis-associated HOD should therefore integrate virological status, fibrosis severity, antiviral exposure and baseline host risk, while clearly distinguishing human clinical evidence from experimental mechanistic findings.

### 3.4 Cholestatic disease: bile acid dysregulation

Cholestatic liver diseases, including PBC and biliary atresia, represent high-risk settings for HOD, in which skeletal injury is closely linked to disturbed bile acid metabolism, alterations in the intestinal microbial ecosystem and impaired nutrient absorption. Cholestasis reduces the delivery of bile acids to the intestinal lumen, thereby altering the ecological environment of the gut microbiota and its capacity for bile acid transformation, and may further contribute to intestinal barrier dysfunction and microbial translocation (Sun et al., 2024). Reduced intraluminal bile acid availability can consequently reshape microbial ecology and bile acid metabolism while potentially reinforcing barrier impairment and bacterial translocation. Although mechanistic evidence in humans remains limited, bile-duct-ligation models provide disease-specific experimental evidence of impaired osteoblastogenesis and increased skeletal fragility in cholestatic states (Weinreb et al., 2004). In parallel, insufficient delivery of bile acids to the intestine can impair the absorption of fat-soluble vitamins. Vitamin D deficiency can reduce intestinal calcium absorption and disrupt calcium–phosphate homeostasis, thereby increasing the risk of bone loss, whereas vitamin K deficiency may impair γ-carboxylation of osteocalcin and compromise bone matrix mineralization (Herrmann et al., 2024; Sun et al., 2024). These intestinal and nutritional consequences of cholestasis therefore constitute important intermediate links between liver disease and skeletal injury.

Alterations in bile acid composition and signalling may additionally exert direct effects on bone remodelling. Cholestasis and gut microbial dysbiosis can disrupt the conversion of primary to secondary bile acids and alter downstream bile acid receptor signalling. Bone cells can respond to bile-acid signalling through receptors including FXR and TGR5, providing a potential route through which alterations in the bile-acid pool may influence bone remodelling (Herrmann et al., 2024; Zheng et al., 2025; Hassan Rizvi et al., 2026). In addition to receptor-mediated effects, bilirubin and selected hydrophobic bile acids that accumulate in the circulation during cholestasis may directly compromise bone-cell function. Experimental evidence indicates that these molecules can impair bone-cell viability, inhibit osteoblast proliferation, differentiation and mineralization, and thereby provide a direct mechanistic route from cholestasis to reduced bone formation (Ruiz-Gaspa et al., 2020a). Oxidative stress and organelle dysfunction may contribute further to these inhibitory effects on osteogenesis (Ruiz-Gaspa et al., 2020b).

Overall, the liver–gut–bone relationship in cholestatic liver disease has distinct disease-specific features. Rather than being dominated by a single inflammatory pathway, it appears to reflect the combined effects of disrupted enterohepatic bile acid circulation, alterations in the intestinal microbial ecosystem, impaired absorption of fat-soluble vitamins and direct toxicity of cholestatic metabolites to bone cells, ultimately resulting in suppressed bone formation and increased skeletal fragility. At present, however, human studies primarily support a clinical association between cholestasis and osteoporosis, whereas direct evidence establishing a continuous causal chain from microbial alterations through bile acid signalling to bone-cell injury remains limited (Schmidt et al., 2019; Sun et al., 2024). Therapeutic strategies that reduce cholestasis or restore bile acid homeostasis may therefore have potential skeletal benefits, but whether they can directly reverse HOD through modulation of the gut microbiota or bile acid signalling requires confirmation in longitudinal clinical studies incorporating bone-remodelling and fracture-related outcomes.

### 3.5 Cirrhosis: multisystem amplification

Cirrhosis represents a convergent advanced stage in which multiple disturbances along the liver–gut–bone axis accumulate across different CLD aetiologies. Portal hypertension, intestinal wall oedema and impaired mucosal defence can further disrupt the intestinal barrier, promoting bacterial translocation and the entry of endotoxins into the portal and systemic circulations, with sustained activation of hepatic and systemic pro-inflammatory and pro-fibrotic signalling through pattern-recognition receptors (Guan et al., 2022). Cirrhosis is frequently associated with gut microbial dysbiosis and intestinal barrier dysfunction, which may reinforce microbial translocation, endotoxaemia and systemic inflammation (Philips and Augustine, 2022; Bloom et al., 2023; Buttler et al., 2025). Intestinal abnormalities in cirrhosis should therefore not be viewed as isolated alterations but as components of a continuous process involving barrier disruption, microbial translocation and inflammatory amplification, which provides a persistent systemic inflammatory milieu that may favour osteoclast differentiation and resorptive activity while contributing, together with endocrine and nutritional abnormalities, to impaired osteoblast-mediated bone formation.

As hepatic function deteriorates, bile acid synthesis and enterohepatic circulation become impaired, while microbial metabolic capacity also changes substantially, altering the generation of multiple signalling molecules, including SCFAs, secondary bile acids and tryptophan-derived metabolites (Milosevic et al., 2019; Liu and Yang, 2023). These alterations may further compromise intestinal barrier function and nutrient absorption and may influence bone remodelling through metabolic and immune pathways that regulate osteoclast differentiation and osteoblast function. In parallel, cirrhosis-associated reductions in IGF-1, chronic inflammation, hypogonadism and malnutrition can collectively suppress bone formation, with increased bone resorption superimposed in a subset of patients. Disruption of the hepatic growth hormone–IGF-1 axis is closely associated with loss of bone mass, whereas inflammatory mediators such as TNF-α and IL-6 may promote osteoclastogenesis through nuclear factor kappa B (NF-κB) (Liu et al., 2018). High-resolution peripheral quantitative CT studies further demonstrate concurrent abnormalities in trabecular and cortical bone microarchitecture in patients with cirrhosis (Wakolbinger et al., 2019). Accordingly, cirrhosis-associated HOD should not be reduced to a fixed ‘high-resorption’ or ‘low-formation’ phenotype; rather, the relative contributions of suppressed bone formation and increased resorption are likely to vary according to underlying aetiology, disease stage and individual metabolic context.

Sarcopenia further amplifies skeletal fragility in patients with cirrhosis. Protein–energy malnutrition, a hypercatabolic state and endocrine dysfunction predispose these patients to loss of muscle mass and strength, which not only reduces mechanical loading of bone but may also impair bone formation through altered myokine and sex-hormone signalling (Nishikawa et al., 2024). Low serum testosterone concentrations are closely associated with both sarcopenia and reduced bone mass, suggesting that sex-hormone deficiency may represent an important link between muscle and skeletal deterioration (Deng et al., 2021). Advanced cirrhosis therefore constitutes a convergent state in which portal hypertension, impaired mucosal defence, dysbiosis, malnutrition, sarcopenia, endocrine dysfunction and reduced IGF-1 can act simultaneously to amplify skeletal injury. Human evidence is substantially stronger for the association between advanced CLD and skeletal impairment than for a causal role of the microbiome in mediating this relationship (Jorgensen et al., 2022; Nachef et al., 2024). Cirrhosis should therefore be regarded as a late-stage amplifying condition shared across CLD aetiologies, and skeletal management should address intestinal barrier dysfunction, nutritional status, endocrine abnormalities and sarcopenia in parallel. A complete human causal pathway linking microbiome alterations to skeletal injury in cirrhosis remains to be established (Philips and Augustine, 2022).

### 3.6 Other CLDs: emerging mechanisms

Skeletal injury associated with autoimmune hepatitis (AIH) reflects the combined effects of disease severity, host characteristics and treatment exposure and should not be attributed solely to glucocorticoid therapy. In a study of 211 patients with AIH, age >54 years, glucocorticoid exposure for >90 months, a BMI <23 kg m^-2^ and liver stiffness >8 kPa on transient elastography were each associated with an increased risk of osteoporosis (Schmidt et al., 2020). Large population-based studies have further shown that patients with AIH have a higher risk of osteoporotic fractures than matched controls, with both cirrhosis and prolonged glucocorticoid exposure being associated with increased fracture risk (Lim et al., 2024). AIH-associated bone disease is therefore better understood as a form of secondary skeletal injury arising from the combined effects of hepatic fibrosis, chronic inflammation and treatment-related factors. For CLD subtypes with limited evidence, mechanistic claims should be explicitly recognized as indirect. In AIH, for example, experimental studies using polymeric immunoglobulin receptor-deficient mice suggest that intestinal dysbiosis and barrier dysfunction can aggravate hepatic inflammation, but direct skeletal outcomes have not been demonstrated. Such findings support a link between intestinal barrier abnormalities and hepatic inflammation in AIH but are insufficient to establish a complete causal liver–gut–bone pathway.

Hereditary haemochromatosis and Wilson disease can also be accompanied by reduced bone mass, although mechanistic and microbiome-related evidence is considerably more limited than that available for MASLD, ALD and cholestatic liver disease. Among patients with hereditary haemochromatosis, the prevalence of osteoporosis and osteopenia has been estimated at approximately 25% and 41%, respectively, and BMD has been associated with iron burden, low BMI, hypogonadism and menopausal status (Valenti et al., 2009). Iron overload may exert direct effects on bone cells, but cirrhosis, diabetes, hypogonadism and other metabolic or endocrine abnormalities may also contribute to skeletal loss; direct iron toxicity should therefore be distinguished from secondary mechanisms (Baschant et al., 2022). Systematic reviews and meta-analyses of Wilson disease similarly suggest increased risks of osteopenia and osteoporosis, although the available studies are generally small and heterogeneous, and recent cohort data indicate that age, sex and musculoskeletal symptoms may influence osteoporosis risk (Chenbhanich et al., 2018; Zhu et al., 2025). At present, there is insufficient evidence to conclude that copper deposition causes bone loss through a defined bone-cell pathway, and continuous human evidence implicating the gut microbiota in Wilson disease-associated skeletal injury is also lacking. These disorders are therefore better considered evidence-limited extensions of the proposed liver–gut–bone axis.

For CLD subtypes in which direct evidence is sparse, experimental models outside the context of liver disease can provide biological plausibility but should not be interpreted as disease-specific evidence. Trimethylamine N-oxide (TMAO), for example, illustrates how a microbiota-derived metabolite may exert direct effects on bone cells. In murine osteoporosis models, excessive TMAO impaired osteogenic differentiation and mineralized matrix formation through a PERK–ATF5-dependent pathway involving endoplasmic-reticulum/mitochondrial stress responses; however, these findings are not specific to CLD (Lin et al., 2024). Similarly, indole-3-acetic acid (IAA) suppressed RANKL-induced PI3K–AKT signalling, reactive oxygen species production and NFATc1 nuclear translocation in osteoclast precursors, and IAA supplementation attenuated bone loss in ovariectomized mice. This evidence derives from non-CLD osteoporosis models and should therefore be interpreted as support for biological plausibility rather than as direct proof of a liver–gut–bone axis in CLD (Bai et al., 2026). Accordingly, in evidence-limited conditions such as AIH, hereditary haemochromatosis and Wilson disease, human clinical associations, disease-specific animal evidence and findings from non-CLD osteoporosis models should be clearly distinguished to avoid extrapolating experimental mechanisms into established causal liver–gut–bone relationships (**Table 2**).

**Table 2:** Disease-specific evidence relevant to the liver–gut–bone axis and degree of mechanistic support.

CLD subtype	Evidence source	Upstream component	Skeletal component	Axis interpretation	Ref
MASLD	MASH mice + separate human bone study	Reduced secondary bile acids via a host hepatic mechanism	Higher FIB-4 linked to lower BMD/TBS	Separate evidence; full axis unproven	(Barchetta et al., 2023; Gillard et al., 2024)
Alcohol-associated liver disease	Human microbiome study + ethanol-exposed mice	Microbiome/metabolites differed across ALD stages	Ethanol suppressed osteoblast- and promoted osteoclast-related signals	Separate gut/metabolite and bone evidence; mediation unproven	(Denys et al., 2022; Ganesan et al., 2024)
Chronic HBV	Human microbiome/metabolomics + FMT mice + separate bone study	Dysbiosis linked to HBV severity; FMT aggravated fibrosis	Higher HBV DNA linked to lower BMD	Liver–gut and bone evidence remain separate	(Shen et al., 2023; Wang et al., 2025; Zhou et al., 2026)
Cholestatic liver disease	PSC patients + Abcb4-deficient mice + bone-cell studies	Th17/IL-17 and cholestatic metabolites	Increased resorption and impaired bone-cell function	Liver/immune/metabolite–bone evidence; gut link unproven	(Schmidt et al., 2019; Ruiz-Gaspa et al., 2020a; Jurado et al., 2022)
Cirrhosis	Human cirrhosis gut/barrier studies + separate cirrhosis bone studies	Dysbiosis, increased intestinal permeability and systemic inflammation	Increased bone turnover, impaired bone microarchitecture and vertebral fractures	Human associations; microbiome-mediated bone injury unproven	(Jorgensen et al., 2022; Ogiso et al., 2022; Philips and Augustine, 2022; Bloom et al., 2023; Buttler et al., 2025)

## 4 Shared and disease-specific mechanisms of the liver–gut–bone axis

### 4.1 Shared downstream mechanisms

MASLD, alcohol-associated liver disease, viral hepatitis and cholestatic liver disease differ substantially in their initiating events, metabolic context and treatment-related modifiers. Despite this aetiological heterogeneity, several downstream disturbances recur as liver disease progresses. Intestinal barrier impairment and microbial translocation can amplify hepatic and systemic inflammatory signalling (Pabst et al., 2023), while pro-inflammatory mediators such as TNF-α and IL-6 may enhance RANKL-dependent osteoclast differentiation and activity while suppressing osteoblast differentiation and bone-forming function (Walsh et al., 2018; Tsukasaki and Takayanagi, 2019; You et al., 2025). This inflammatory convergence is particularly evident in ALD and advanced cirrhosis, in which barrier dysfunction and microbial translocation are prominent. In MASLD, similar inflammatory pathways coexist with obesity, insulin resistance and adipokine dysregulation, whereas in cholestatic disease they occur alongside disturbances in bile acid homeostasis and fat-soluble vitamin absorption.

In advanced CLD, skeletal impairment is also closely associated with endocrine dysfunction, nutritional deficiency and loss of muscle mass. The liver is an important source of circulating IGF-1, and disruption of the hepatic growth hormone–IGF-1 axis has been associated with impaired skeletal integrity in chronic liver disease (Liu et al., 2018). In addition, reduced IGF-1 frequently coexists with hypogonadism, protein–energy malnutrition, vitamin D insufficiency and sarcopenia, together compromising osteoblast function, mechanical loading and resistance to falls and fractures (Liu et al., 2018; Warner Ii and Satapathy, 2023). Their relative importance, however, varies by aetiology. Fat-soluble vitamin malabsorption is particularly relevant in cholestatic disease, metabolic comorbidities such as obesity and type 2 diabetes modify skeletal risk in MASLD, and ALD combines nutritional and endocrine abnormalities with direct ethanol-related osteotoxicity. These disease-specific disturbances ultimately affect a common set of skeletal processes, although the balance between impaired bone formation and increased bone resorption differs according to disease subtype and stage. Cirrhosis is associated with altered bone turnover and deterioration of both trabecular and cortical microarchitecture, whereas experimental evidence in cholestatic disease particularly supports impaired osteoblastogenesis. HOD should therefore be regarded as a heterogeneous disorder of bone remodelling rather than as a uniformly high-turnover or low-turnover state (**Figure 1**).

**Figure 1:**
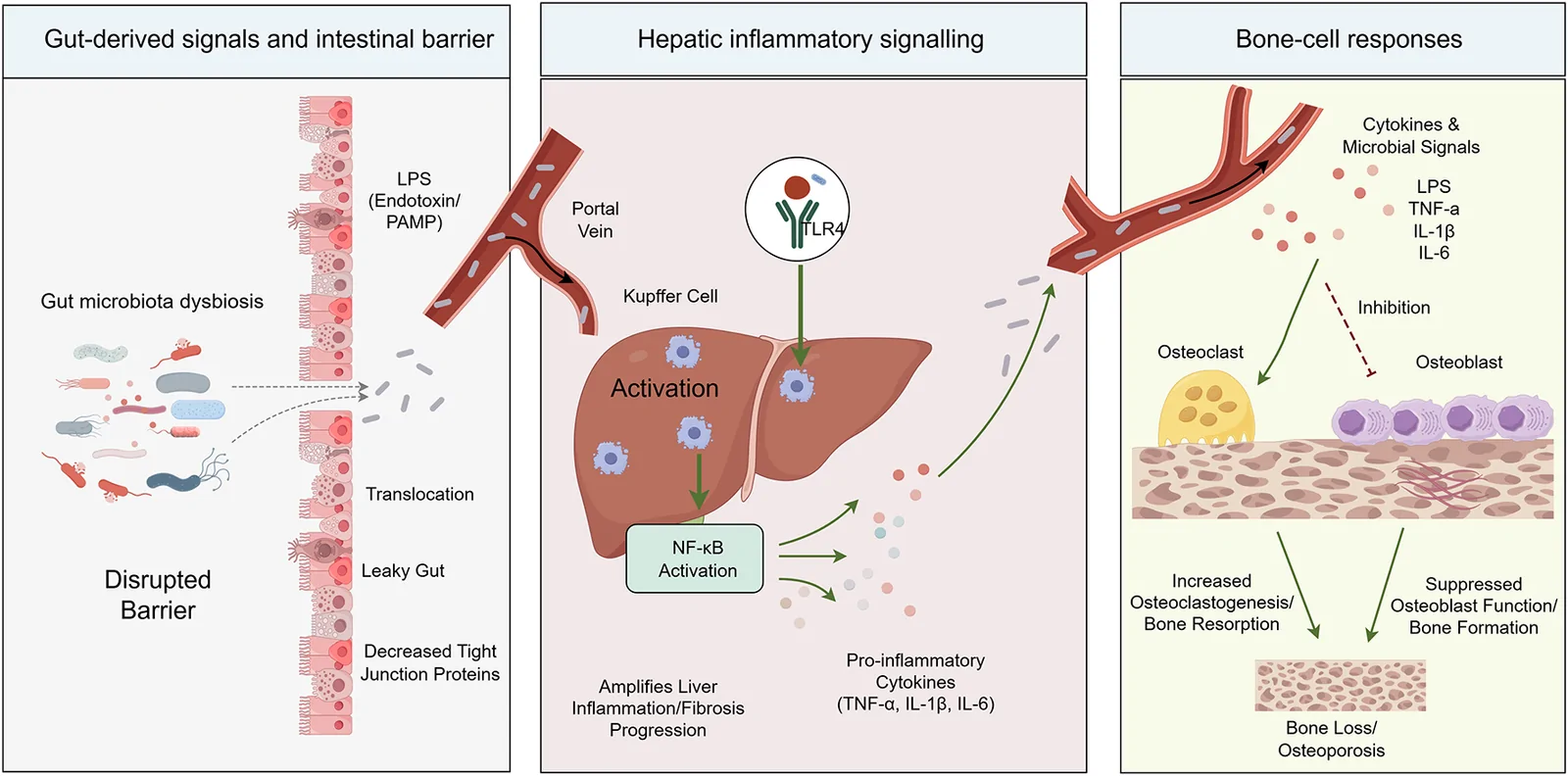
Molecular and cellular mechanisms linking gut-derived signals to impaired bone remodelling in chronic liver disease. Gut microbiota dysbiosis and intestinal barrier disruption facilitate the translocation of microbial-associated molecular patterns, particularly lipopolysaccharide (LPS), into the portal and systemic circulation. Gut-derived signals activate hepatic innate immune pathways, including TLR4–NF-κB signalling in Kupffer cells, and promote the release of pro-inflammatory cytokines such as TNF-α, IL-1β and IL-6. These inflammatory mediators may enhance osteoclastogenesis and bone resorption while suppressing osteoblast function and bone formation, thereby disrupting osteoblast–osteoclast coupling and contributing to bone loss and osteoporosis.

### 4.2 Disease-specific upstream drivers

The principal upstream drivers of skeletal injury differ according to CLD aetiology. In MASLD, metabolic dysfunction, insulin resistance and adipokine imbalance are dominant modifiers, with dysregulation of leptin and adiponectin and chronic low-grade inflammation further perturbing bone remodelling. TLR4-related inflammatory signalling and mediators such as TNF-α and IL-6 may promote osteoclastogenesis while suppressing osteoblast function (Zhu et al., 2024; Bagherifard et al., 2025). By contrast, ALD combines direct alcohol-induced osteotoxicity with nutritional deficiency and endocrine dysfunction. Cholestasis can be accompanied by abnormalities in fat-soluble vitamin metabolism, including vitamins D and K, which may contribute to disturbances in calcium homeostasis and bone mineralisation (Gasser, 2008; Pugliese et al., 2022). Retained hydrophobic bile acids and other cholestasis-associated molecules may additionally suppress osteoblast differentiation and function (Ruiz-Gaspa et al., 2020b), while reduced intestinal bile acid availability can alter gut microbial composition and metabolic activity and may further influence systemic inflammation and bone metabolism through effects on barrier integrity, immune regulation and microbial metabolite production (Sun et al., 2024). Cholestatic disease therefore represents a distinct upstream context in which altered bile acid homeostasis, nutrient malabsorption and direct osteoblast toxicity coexist.

In viral hepatitis, skeletal injury reflects the combined effects of chronic infection, hepatic dysfunction and antiviral exposure. In chronic HCV infection, systemic inflammatory activity has been associated with abnormal bone microarchitecture, although current human evidence is insufficient to establish inflammation as an independent causal pathway directly leading to osteoporosis (Weinstein et al., 2025). Treatment-related effects are supported by clearer clinical evidence: adefovir-associated proximal renal tubular injury can cause phosphate wasting, hypophosphataemia and osteomalacia (Park et al., 2018), while long-term TDF therapy requires attention to renal and skeletal safety. Tenofovir alafenamide has been associated with smaller reductions in hip and spine BMD than TDF in randomised controlled trials (Buti et al., 2024). In HCV infection, viral eradication can reduce systemic inflammation without necessarily producing parallel improvements in BMD, and current evidence remains insufficient to establish the gut microbiota as a mediator of this process (Psomas et al., 2025). Collectively, these findings indicate that the upstream determinants of skeletal injury remain strongly aetiology-specific, whereas their downstream consequences partially converge on enhanced inflammation, impaired bone formation and increased bone resorption.

### 4.3 Cirrhosis as an amplifier of skeletal injury

Cirrhosis represents a common advanced stage of multiple chronic liver diseases and can progressively attenuate aetiology-specific upstream differences by generating a more convergent inflammatory, metabolic and catabolic milieu, thereby amplifying skeletal injury. Rather than representing an independent disease-specific mechanism, cirrhosis should be considered a stage-dependent amplifier that intensifies pre-existing abnormalities across different CLD aetiologies. Advanced cirrhosis integrates multiple risk domains, including inflammation, intestinal barrier dysfunction, endocrine disturbance, malnutrition and sarcopenia, thereby increasing skeletal vulnerability. Importantly, current human evidence is stronger for the association between cirrhosis severity and skeletal impairment than for a causal role of microbiota-mediated mechanisms. Cirrhosis may therefore establish a skeletal-damaging environment in which intestinal barrier dysfunction, microbial translocation and inflammatory amplification act in concert (Hsu and Schnabl, 2023). However, studies simultaneously assessing intestinal barrier integrity, microbiota and metabolite alterations, and bone-cell endpoints within the same cirrhotic population remain scarce. Consequently, this mechanistic continuum should currently be regarded as a biologically plausible model rather than a fully validated causal pathway in humans (Xia et al., 2023).

Beyond inflammatory mechanisms, endocrine, nutritional and muscular abnormalities associated with advanced hepatic dysfunction can further impair bone formation and increase susceptibility to fracture. Reduced hepatic IGF-1 production, hypogonadism, protein–energy malnutrition and sarcopenia frequently coexist. Low IGF-1 diminishes anabolic support for osteoblasts, whereas loss of muscle mass and strength can exacerbate skeletal fragility by reducing mechanical loading on bone and increasing frailty and the risk of falls (Liu et al., 2018; Warner Ii and Satapathy, 2023). Impaired hepatic synthetic function may also alter circulating concentrations of albumin and vitamin D-binding protein, although such changes should not be interpreted simply as evidence of impaired bone-matrix synthesis. In patients with decompensated cirrhosis in particular, calcium, phosphate and vitamin D status may additionally be influenced by renal dysfunction, nutritional status and electrolyte disturbances and should therefore be interpreted alongside 25(OH)D concentrations, renal function and clinical nutritional assessment. Cirrhosis thus does not produce a single, fixed bone-turnover phenotype; rather, interacting inflammatory, endocrine, nutritional and sarcopenic abnormalities collectively amplify skeletal risk across CLD aetiologies.

The peri-transplant period further illustrates the clinical consequences of this amplification of skeletal risk. Many liver transplant candidates already have low BMD or impaired bone microarchitecture before transplantation, and additional factors in the early post-transplant period—including glucocorticoids and other immunosuppressive agents, reduced mobility and inadequate nutritional intake—can contribute to further bone loss (Lim et al., 2021). Glucocorticoids are particularly important contributors to early post-transplant skeletal loss through suppression of osteoblast function and enhancement of bone resorption, whereas immobilisation and sarcopenia further reduce mechanical stimulation of the skeleton. Cirrhosis and the peri-transplant period can therefore be regarded as phases of particularly concentrated risk for CLD-associated bone disease. Clinically, this framework not only helps to explain the convergence of skeletal injury across different liver disease aetiologies but also supports systematic assessment of BMD, fracture risk, nutritional status and sarcopenia before and after liver transplantation, together with the early implementation of targeted bone-protective strategies.

## 5 Clinical management and therapeutic translation

### 5.1 Standard-of-care management

Bone health management in patients with CLD should be guided by stratified assessment according to liver disease subtype, disease stage and conventional fracture risk factors. Baseline DXA should be considered in patients at increased skeletal risk, including those with cirrhosis, cholestasis, a history of fragility fracture, prolonged glucocorticoid exposure, postmenopausal status, advanced age or low body weight, with the timing of follow-up determined by baseline BMD, disease progression and treatment exposure (European Association for the Study of the L., 2019). Baseline evaluation should also document fragility fractures, falls, height loss, back pain, glucocorticoid exposure, alcohol use and reduced physical activity. BMD should be assessed at the lumbar spine and hip, and vertebral fracture assessment or spinal imaging should be considered when occult vertebral compression fracture is suspected, particularly in patients with height loss, kyphosis or persistent back pain (European Association for the Study of the L., 2019). Management should additionally address calcium and vitamin D deficiency, malnutrition, hypogonadism, excessive alcohol consumption and physical inactivity. Vitamin D supplementation should be individualised according to serum 25(OH)D, calcium and phosphate concentrations, renal function, absorptive capacity and the underlying liver disease. Particular attention should be paid to fat-soluble vitamin malabsorption in cholestatic disease, whereas routine use of active vitamin D analogues is not warranted in all patients with CLD.

For patients who meet criteria for pharmacological treatment, bisphosphonates remain widely used first-line antiresorptive agents. Oral alendronate can be considered in patients able to tolerate oral therapy, whereas intravenous zoledronic acid may be appropriate when gastrointestinal intolerance, malabsorption or poor adherence limits oral treatment. Renal function, serum calcium and other relevant electrolyte abnormalities should be assessed before and during therapy. Denosumab inhibits bone resorption by blocking RANKL-dependent osteoclast formation and activation and may provide an alternative for selected patients in whom bisphosphonates are unsuitable or poorly tolerated. However, its discontinuation is associated with rapid bone loss and an increased risk of vertebral fractures; consequently, a planned sequential antiresorptive strategy is required when denosumab is stopped (Cosman et al., 2022). Treatment selection should therefore not be based on hepatic function alone, but should incorporate previous fragility fractures, BMD, renal function, the risk of hypocalcaemia and upper gastrointestinal comorbidities.

In patients at very high fracture risk, with severe osteoporosis or with established fragility fractures, anabolic therapy such as teriparatide may be considered after careful assessment of hepatic and renal function, serum calcium and relevant contraindications. The use of romosozumab should be individualised in accordance with current osteoporosis guidelines and the patient’s cardiovascular risk profile (Qaseem et al., 2023). Sequential antiresorptive therapy is generally required after completion of an anabolic regimen to preserve gains in bone mass. More broadly, selection of anti-osteoporotic therapy should integrate previous fragility fractures, BMD, estimated fracture probability, renal function, hypocalcaemia risk, upper gastrointestinal comorbidity and cardiovascular risk (Reid and Billington, 2022). Accordingly, standard management of CLD-associated HOD should be founded on fracture-risk stratification, optimisation of nutrition and lifestyle, and evidence-based anti-osteoporotic therapy. Microbiota- or metabolite-targeted interventions remain exploratory and should currently be regarded as potential adjuncts rather than replacements for established standard care.

### 5.2 Disease-specific management

Bone health management across CLD subtypes should combine standard osteoporosis care with interventions directed at the dominant aetiology-specific risk factors. In MASLD, dietary modification, regular physical activity and optimisation of metabolic risk factors remain central to management and can improve hepatic steatosis and insulin resistance (European Association for the Study of the L. et al., 2024). However, weight-loss strategies should avoid excessive restriction of energy and protein intake, which may exacerbate malnutrition and bone loss. Available studies suggest that the association between MASLD and low BMD is attenuated after adjustment for confounding factors such as obesity, indicating that skeletal risk cannot be attributed to hepatic steatosis alone (Ciardullo et al., 2021). By contrast, the association between hepatic fibrosis and reduced BMD appears more consistent, supporting closer skeletal surveillance in patients with more advanced disease (Yoon et al., 2021; Barchetta et al., 2023). In alcohol-related liver disease, sustained abstinence remains a central aetiological intervention and should be accompanied by correction of protein, calcium and vitamin D deficiencies and assessment of associated hypogonadism and sarcopenia (Luo et al., 2017). Established osteoporosis should nevertheless be assessed and treated independently according to fracture risk (Eastell et al., 2019).

Management of cholestatic liver disease should address both the underlying hepatic disorder and skeletal protection. In PBC, for example, ursodeoxycholic acid (UDCA) is an important disease-modifying treatment, but improvement in cholestasis should not be regarded as sufficient treatment for established osteoporosis. Skeletal risk should still be stratified according to DXA findings, previous fracture history and calcium and vitamin D status, with particular attention to fat-soluble vitamin malabsorption and malnutrition (You et al., 2022). Abnormal sclerostin expression has been reported in chronic cholestasis and may contribute to impaired bone formation (Guanabens et al., 2016). However, evidence remains insufficient to recommend sclerostin-targeted or vitamin K–based therapy specifically for PBC-associated osteoporosis, and treatment should currently follow established osteoporosis management principles. Management should therefore combine treatment of the primary liver disease with evidence-based osteoporosis therapy rather than prematurely equating mechanistically attractive interventions with established standard care.

Bone health management in viral hepatitis should incorporate viral control, the stage of liver disease and exposure to antiviral therapy. In CHB, TAF and TDF have comparable antiviral efficacy, but two phase III randomised trials demonstrated significantly smaller reductions in hip and spine BMD with TAF than with TDF (Buti et al., 2016; Chan et al., 2016). Accordingly, in patients with pre-existing osteoporosis, renal impairment or otherwise elevated bone–renal risk, nucleos(t)ide analogue selection should follow current guidelines and favour regimens with a more favourable renal and skeletal safety profile where appropriate (European Association for the Study of the L., 2025). In CHC, sustained virological response after direct-acting antiviral (DAA) therapy substantially reduces systemic inflammation, yet short-term improvements in BMD or trabecular bone score do not necessarily occur in parallel (Psomas et al., 2025). Thus, neither long-term HBV suppression nor virological cure of HCV obviates the need for independent fracture-risk assessment and, where indicated, osteoporosis treatment. The central principle of disease-specific management is therefore not to construct an entirely separate skeletal treatment algorithm for each CLD subtype, but to identify and correct aetiology- and treatment-related risk factors within a common framework of evidence-based bone protection.

### 5.3 Microbiota- and metabolite-targeted therapies

Microbiota-targeted strategies, including probiotics, prebiotics, dietary fibre and selected dietary patterns, have the potential to improve CLD-associated intestinal dysbiosis, but direct clinical evidence for skeletal benefit remains lacking. Current studies primarily indicate that these interventions can alter microbial community composition, enhance intestinal barrier function or improve hepatic metabolic phenotypes (Hsu and Schnabl, 2023); trials simultaneously evaluating BMD, bone microarchitecture and fracture outcomes in patients with CLD remain scarce. Improvements in hepatic or microbiological endpoints therefore cannot be assumed to confer skeletal protection. Recent interest has focused on manipulating gut microbial communities and their metabolic outputs, although the available evidence remains predominantly mechanistic, preclinical or indirect. Dietary fibre, prebiotics and selected dietary patterns may increase SCFA production, reinforce tight-junction integrity and modulate the regulatory T cell (Treg)–Th17 cell axis, but whether these effects reduce fracture risk in CLD has not been established. Studies in non-CLD models of osteoporosis further suggest that SCFAs can suppress osteoclast differentiation and attenuate inflammation-associated bone loss, providing biological support for a gut microbiota–bone remodelling connection; however, its clinical relevance requires validation in well-phenotyped CLD populations stratified by aetiology and disease stage (Yang et al., 2024).

Faecal microbiota transplantation (FMT) provides proof that the intestinal microbiota is clinically modifiable in advanced liver disease, but these findings cannot currently be extrapolated to CLD-associated osteoporosis. Small clinical trials in patients with cirrhosis and recurrent hepatic encephalopathy have shown that FMT using selected donors can improve cognition and microbial dysbiosis and may reduce subsequent hospitalisation, but these studies did not evaluate BMD, bone-turnover markers or fracture outcomes (Bajaj et al., 2017; Bajaj et al., 2025). FMT should therefore be viewed as a clinical proof of concept that the liver–gut microbial interface can be therapeutically manipulated rather than as evidence for treatment of CLD-associated HOD. Future trials will need to prespecify skeletal endpoints, including bone turnover, bone microarchitecture and fracture risk, to determine whether microbiota remodelling translates into genuine skeletal benefit.

Microbial metabolites and their receptor-mediated signalling pathways represent another class of mechanistically informed therapeutic targets, among which the bile acid receptors FXR and TGR5 are particularly relevant to liver disease. Considerable mechanistic evidence supports bidirectional interactions between the gut microbiota and bile acid metabolism in cholestatic liver disease, and bone cells can also respond to bile acid-related signalling. However, no clinical trial specifically conducted in CLD-associated osteoporosis has yet demonstrated that modulation of FXR or TGR5 improves BMD or reduces fracture risk, and evidence for TGR5-related skeletal effects remains largely derived from cellular and animal models (Zheng et al., 2025). Future studies should therefore simultaneously assess microbial composition, bile acid profiles, receptor signalling, bone turnover and clinically relevant skeletal outcomes in patients stratified by CLD aetiology and stage, rather than extrapolating skeletal effects from isolated hepatic or intestinal endpoints. Overall, microbiota- and metabolite-targeted therapies should be positioned as potential adjunctive strategies, not as alternatives to correction of nutritional deficiencies, evidence-based anti-osteoporotic pharmacotherapy and treatment of the underlying liver disease (**Figure 2**).

**Figure 2:**
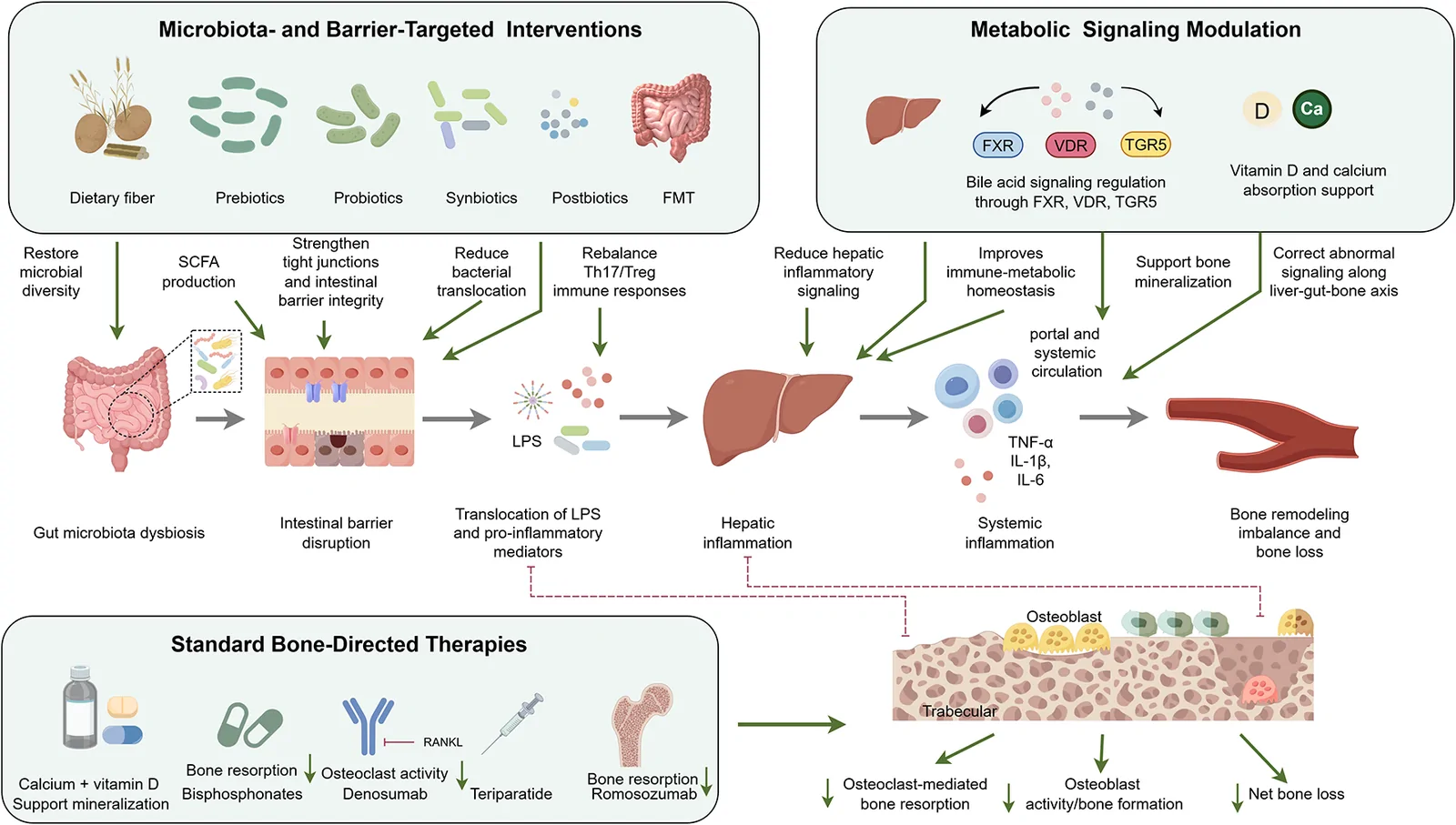
Current and emerging therapeutic strategies for the liver–gut–bone axis in chronic liver disease-associated osteoporosis. Standard care includes treatment of the underlying liver disease, correction of nutritional deficiencies, and anti-osteoporotic therapy. Microbiota-targeted approaches remain investigational and should be viewed as complementary rather than alternative therapies. Green arrows indicate therapeutic effects, whereas dashed red lines indicate disease-promoting pathways.

### 5.4 Priorities for future clinical trials

Future research on CLD-associated HOD should move beyond cross-sectional associations towards prospective, stratified studies designed to test mechanisms and temporal relationships. Study populations should be prospectively stratified according to CLD aetiology, fibrosis stage and compensated versus decompensated disease, with systematic documentation of major confounders including age, sex and menopausal status, BMI, diabetes, alcohol exposure, nutritional status, sarcopenia and relevant medications. Skeletal outcomes should include, at a minimum, DXA-derived BMD, vertebral fracture assessment and standardised bone-turnover markers, with trabecular bone score and measures of bone microarchitecture incorporated whenever feasible. Trial protocols should additionally prespecify factors such as obesity, diet, geographical background and microbiome methodology. Ideally, microbiome and metabolomic measurements should be acquired contemporaneously with BMD, bone microarchitecture, bone-turnover markers and fracture outcomes, thereby enabling mediation and causal relationships to be evaluated within the same dataset rather than inferred from separate studies. Such designs will be essential for distinguishing aetiology-specific skeletal effects from the common consequences of advanced liver disease.

Pivotal randomised controlled trials should place greater emphasis on clinically relevant outcomes, particularly fractures, rather than relying exclusively on BMD as a surrogate endpoint, because vertebral fractures can occur in patients with CLD—especially those with cirrhosis—even when BMD has not crossed the conventional threshold for osteoporosis (Nachef et al., 2024). Future trials should evaluate the efficacy and safety of established osteoporosis therapies, including bisphosphonates and denosumab, across defined CLD subtypes and disease stages, with prespecified analyses of how renal function, calcium–phosphate homeostasis and hepatic functional status modify treatment efficacy and adverse events. By contrast, FMT, probiotics, synbiotics, postbiotics, dietary fibre and metabolite-targeted approaches should be explicitly classified as exploratory interventions. Trials of these strategies should rigorously document factors that alter the intestinal microbiota—including diet, antibiotics, proton-pump inhibitors, lactulose and rifaximin—and should obtain contemporaneous microbiome, metabolomic, inflammatory and skeletal measurements. Establishing the temporal sequence between microbial alterations, metabolite changes and subsequent changes in bone remodelling within the same patient cohort will be critical to strengthening causal inference for the liver–gut–bone axis.

Multilevel combination strategies and biomarker validation represent important priorities for the next phase of research. In selected CLD subtypes, particularly cholestatic liver diseases, studies could explore potential synergy between treatment of the primary liver disease, modulation of the intestinal microbiota and conventional osteoporosis therapy. In PBC, for example, interventions acting at different levels—including UDCA, probiotics and bisphosphonates—could be evaluated using factorial designs capable of separating their independent and interactive effects. Bone-turnover markers and circulating microbial-derived metabolites, including SCFAs and bile acids, may also serve as exploratory biomarkers, but they cannot yet be regarded as validated ‘liquid biopsies’ of skeletal disease. For example, impaired suppression of CTX after an oral glucose load in patients with NAFLD and type 2 diabetes suggests that postprandial regulation of bone remodelling may be disturbed (Maagensen et al., 2018). However, such biomarkers are sensitive to diet, medication exposure, hepatic and renal clearance, and analytical platform. Prospective multicentre studies should therefore determine whether these measures provide predictive information beyond BMD, bone microarchitecture and established bone-turnover markers, with fractures and other clinically meaningful skeletal outcomes serving as key validation endpoints.

## 6 Conclusions

Hepatic osteodystrophy is an important complication of chronic liver disease, but its skeletal phenotypes and upstream drivers vary substantially across CLD subtypes. In MASLD, skeletal risk is closely linked to metabolic dysfunction and the severity of hepatic fibrosis; ALD combines the direct osteotoxic effects of alcohol with malnutrition; and cholestatic liver diseases are distinguished by disrupted bile acid homeostasis and malabsorption. Cirrhosis, as a common advanced stage of multiple CLDs, can further amplify skeletal injury through portal hypertension, sarcopenia, endocrine dysfunction and nutritional abnormalities. The liver–gut–bone axis provides a unifying conceptual framework through which these heterogeneous mechanisms can be integrated. By synthesising current evidence across chronic liver disease, gut microbial dysbiosis and osteoporosis, this review identifies the gut microbiota and its metabolites as potential regulators within this axis. However, most available evidence is derived from cross-sectional studies, animal models or non-CLD osteoporosis systems and is therefore insufficient to establish a complete causal pathway in humans. Microbial signatures also vary substantially according to CLD subtype, disease stage, geography, diet, medications and analytical methodology. Accordingly, variables such as insulin resistance, cumulative alcohol exposure, bile acid profiles and specific microbial features are currently better suited to research stratification than to routine clinical decision-making and should not replace conventional risk assessment based on fragility fracture history, DXA-derived BMD, age, sex and menopausal status, nutritional status, medication exposure and liver disease severity.

Future research should move from descriptive association towards longitudinal, mechanistically informed studies stratified by disease aetiology and stage. Microbial composition, metabolite profiles, immuno-inflammatory pathways, bone-turnover markers, bone microarchitecture and fracture outcomes should be evaluated contemporaneously within the same patient cohorts to identify which components of the liver–gut–bone axis are truly disease-specific and which have causal relevance. Cirrhosis should be conceptualised as a stage-dependent amplifier superimposed on distinct aetiological backgrounds, whereas interventions targeting the microbiome, bile acid receptors or microbial metabolites should remain exploratory adjuncts to, rather than replacements for, established osteoporosis therapy. Future studies should identify disease-specific microbial signatures, metabolite profiles and immuno-inflammatory pathways that predict bone loss and fracture risk and validate them in longitudinal, well-stratified human cohorts. **Figure 3** provides an integrated synthesis of this framework: distinct CLD-specific upstream drivers converge on shared downstream disturbances in osteoblast–osteoclast coupling, with cirrhosis acting as a stage-dependent amplifier. Ultimately, the goal should be to establish a stratified management framework integrating control of the underlying liver disease, mechanistic insights into the intestinal microbiota and targeted skeletal therapy, thereby providing a stronger evidence base for precision risk assessment and individualised intervention in CLD-associated HOD.

**Figure 3:**
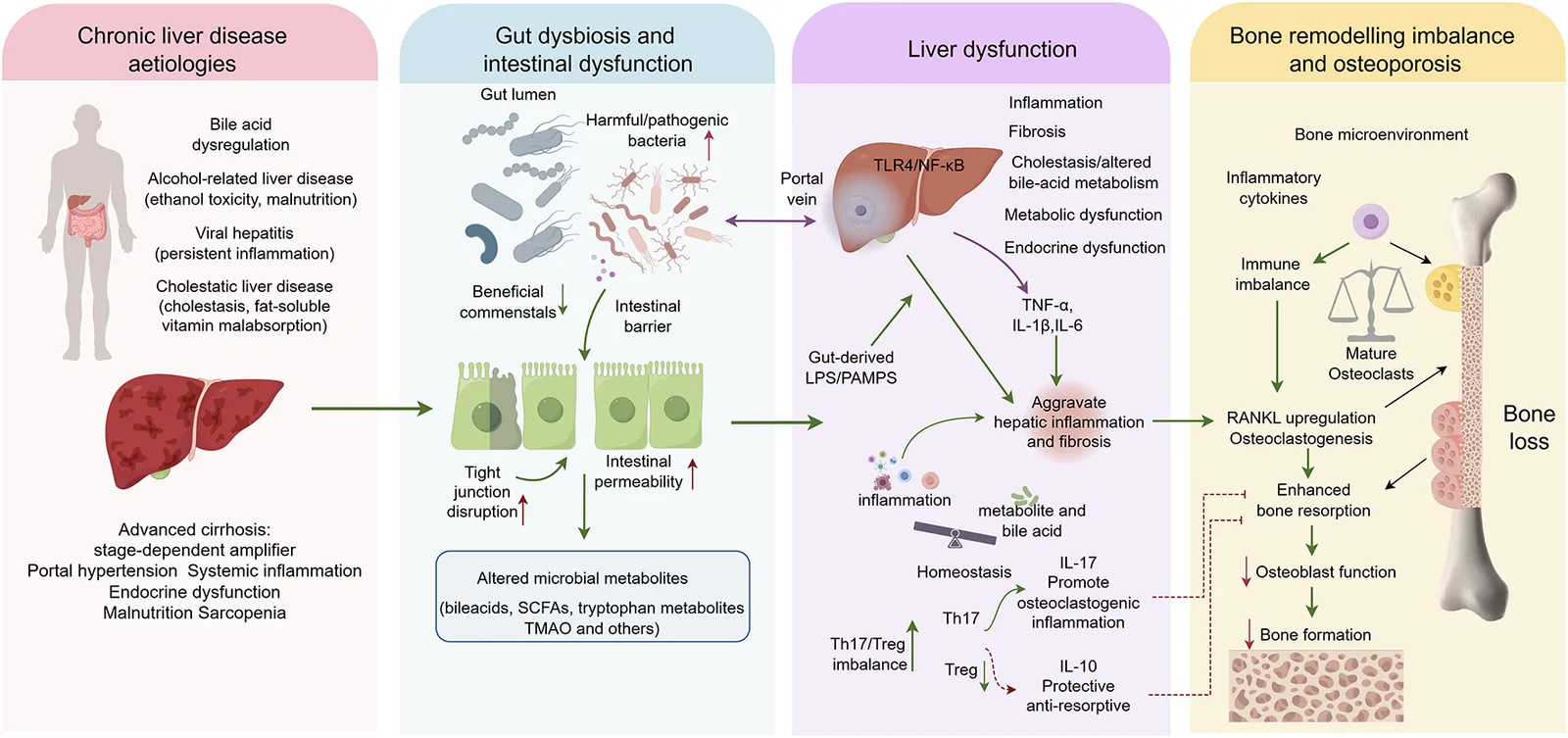
Organ-level overview of the liver–gut–bone axis in chronic liver disease-associated osteoporosis. Distinct chronic liver disease aetiologies are associated with gut dysbiosis, intestinal barrier dysfunction and altered microbial metabolites, which interact with hepatic inflammation, fibrosis, cholestasis and metabolic or endocrine dysfunction. Gut- and liver-derived microbial products, metabolites and inflammatory signals may enter the systemic circulation and contribute to disturbed bone remodelling through immune, metabolic and nutritional pathways. These cross-organ interactions can promote increased bone resorption and impaired bone formation, ultimately leading to bone loss and osteoporosis. Advanced cirrhosis acts as a stage-dependent amplifier through portal hypertension, systemic inflammation, endocrine dysfunction, malnutrition and sarcopenia.

## Glossary

25(OH)D: 25-hydroxyvitamin D
AIH: autoimmune hepatitis
ALD: alcohol-associated liver disease
BMD: bone mineral density
BMI: body mass index
CHB: chronic hepatitis B
CHC: chronic hepatitis C
CLD: chronic liver disease
CT: computed tomography
CTX: C-terminal telopeptide of type I collagen
DAA: direct-acting antiviral
DXA: dual-energy X-ray absorptiometry
FIB-4: Fibrosis-4 Index
FMT: faecal microbiota transplantation
FRAX: Fracture Risk Assessment Tool
FXR: farnesoid X receptor
HBV: hepatitis B virus
HCV: hepatitis C virus
HIV: human immunodeficiency virus
HOD: hepatic osteodystrophy
HR-pQCT: high-resolution peripheral quantitative computed tomography
IAA: indole-3-acetic acid
IGF-1: insulin-like growth factor 1
IL-6: interleukin-6
IL-17: interleukin-17
LPS: lipopolysaccharide
MASLD: metabolic dysfunction-associated steatotic liver disease
MASH: metabolic dysfunction-associated steatohepatitis
NAFLD: non-alcoholic fatty liver disease
NF-κB: nuclear factor kappa B
NFATc1: nuclear factor of activated T cells 1
OPG: osteoprotegerin
P1NP: procollagen type I N-terminal propeptide
PBC: primary biliary cholangitis
PI3K: phosphoinositide 3-kinase
PSC: primary sclerosing cholangitis
RANK: receptor activator of nuclear factor kappa B
RANKL: receptor activator of nuclear factor kappa B ligand
SCFAs: short-chain fatty acids
SHBG: sex hormone-binding globulin
SVR: sustained virological response
TAF: tenofovir alafenamide
TBS: trabecular bone score
TDF: tenofovir disoproxil fumarate
TGR5: Takeda G protein-coupled receptor 5
Th17: T helper 17
TLR4: Toll-like receptor 4
TMAO: trimethylamine N-oxide
TNF-α: tumour necrosis factor alpha
TRAcP5b: tartrate-resistant acid phosphatase 5b
Treg: regulatory T cell
UDCA: ursodeoxycholic acid.
